# Characterization of the Interaction between the Cohesin Subunits Rad21 and SA1/2

**DOI:** 10.1371/journal.pone.0069458

**Published:** 2013-07-12

**Authors:** Nenggang Zhang, Yunyun Jiang, Qilong Mao, Borries Demeler, Yizhi Jane Tao, Debananda Pati

**Affiliations:** 1 Texas Children' Cancer Center, Department of Pediatric Hematology/Oncology, Baylor College of Medicine, Houston, Texas, United States of America; 2 Department of Biochemistry and Cell Biology, Rice University, Houston, Texas, United States of America; 3 Department of Biochemistry, University of Texas Health Science Center, San Antonio, Texas, United States of America; Virginia Tech, United States of America

## Abstract

The cohesin complex is responsible for the fidelity of chromosomal segregation during mitosis. It consists of four core subunits, namely Rad21/Mcd1/Scc1, Smc1, Smc3, and one of the yeast Scc3 *orthologs SA1* or *SA2*. Sister chromatid cohesion is generated during DNA replication and maintained until the onset of anaphase. Among the many proposed models of the cohesin complex, the 'core' cohesin subunits Smc1, Smc3, and Rad21 are almost universally displayed as tripartite ring. However, other than its supportive role in the cohesin ring, little is known about the fourth core subunit SA1/SA2. To gain deeper insight into the function of SA1/SA2 in the cohesin complex, we have mapped the interactive regions of SA2 and Rad21 in vitro and ex vivo. Whereas SA2 interacts with Rad21 through a broad region (301–750 aa), Rad21 binds to SA proteins through two SA-binding motifs on Rad21, namely N-terminal (NT) and middle part (MP) SA-binding motif, located at 60–81 aa of the N-terminus and 383–392 aa of the MP of Rad21, respectively. The MP SA-binding motif is a 10 amino acid, α-helical motif. Deletion of these 10 amino acids or mutation of three conserved amino acids (L^385^, F^389^, and T^390^) in this α-helical motif significantly hinders Rad21 from physically interacting with SA1/2. Besides the MP SA-binding motif, the NT SA-binding motif is also important for SA1/2 interaction. Although mutations on both SA-binding motifs disrupt Rad21-SA1/2 interaction, they had no apparent effect on the Smc1-Smc3-Rad21 interaction. However, the Rad21-Rad21 dimerization was reduced by the mutations, indicating potential involvement of the two SA-binding motifs in the formation of the two-ring handcuff for chromosomal cohesion. Furthermore, mutant Rad21 proteins failed to significantly rescue precocious chromosome separation caused by depletion of endogenous Rad21 in mitotic cells, further indicating the physiological significance of the two SA-binding motifs of Rad21.

## Introduction

DNA replication in the S-phase produces two identical copies of the chromosomal DNA, called sister chromatids. The sister chromatids are held together until the metaphase-to-anaphase transition, when they are segregated to opposite poles. Holding the sister chromatids together, or cohesion, is accomplished by a multi-protein, ring-like complex called cohesin. Cohesin is composed of four core subunits, Smc1, Smc3, Rad21, and the stromal antigen protein SA1 or SA2 [1]–[3]. Smc1 and Smc3 are rod-shaped proteins containing ATP-binding cassette (ABC)-like ATPase motifs. They fold into intramolecular anti-parallel coiled coils and jointly form a V-shaped Smc1/Smc3 heterodimer through their hinge domains [4]-[6]. The N- and C-termini of each SMC molecule form an ATPase head domain at the distal end of the two coiled-coils arms [5]. Rad21 binds to the ATPase heads of Smc3 and Smc1 via its N- and C- terminus, respectively, resulting in the formation of a contiguous tripartite ring [5]. Besides the four core subunits, several other proteins, Pds5A, Pds5B, Sororin, and Wapl, also associate with the cohesin complex [7]–[12].

In higher eukaryotes, cohesins are removed from chromosomes in two phases, the prophase and the anaphase. In the prophase, most of cohesins are removed from the chromosome arms by Pds5-Wapl complex after the cohesins are phosphorylated by kinase Plk1 [26]–[29]. Recent studies indicate that the cohesin-associated protein Sororin coordinates Plk1-mediated chromosomal arm separation [12], [13]. Calcium-inducible cleavage of Rad21 by Calpain-1 also promotes chromosomal arm separation [14]. At the onset of the anaphase, the centromeric and residual arm cohesins are removed by a Separase-mediated cleavage of Rad21 at residues R^172^ and R^450^ [15], [16], culminating in separation of the sisters [32]–[34].

Based on the molecular associations of cohesin subunits, we have provided evidence for a handcuff model of the cohesin complex, which consists of two rings [17], [18]. Each ring has one set of Rad21, Smc1, and Smc3 molecules. The handcuff is established after two Rad21 molecules are orientated in anti-parallel fashion and enforced by either SA1 or SA2 and potentially other cohesin-associated factors. Sister chromatids are held together by one of the two rings. Failure in the formation and maintenance of sister chromatid cohesion results in premature chromatid segregation, which is thought to be a major pathway to aneuploidy, a characteristic feature of most if not all human cancers [19]. A recent study indicated that the SA2 gene is mutated in a number of human tumors, including glioblastoma and melanoma, and targeted inactivation of SA2 leads to sister chromatid cohesion defects and aneuploidy, suggesting a direct role of SA2 in the development of human cancer [20].

In human, SA2 is more abundant than SA1 [2], and the two do not bind to the same cohesin complex [17]. SA2 is present predominantly in the cohesin complex at the chromosomal arm and centromeric regions, whereas SA1 has been described to be responsible for the telomeric cohesion [21]. SA1 and SA2 share ∼70% sequence identity with the homology, mostly located in the region of 69–1075 aa of SA1. However, the role of SA1/SA2 proteins, other than their association with the tripartite ring and their essential function in maintaining chromosomal cohesion, has not been fully investigated. It remains unclear if SA1/2 is required for the assembly and maintenance of the cohesin ring, or if the physical association of SA1/2 and Rad21 takes place through direct binding or indirect interaction requiring additional cohesin-associated components. Although the region of 362–403 aa of Rad21 has been reported to bind to SA1 [22], the fine mapping of the Rad21-SA1/2 interaction domains and the functional mechanism of SA1/2 in chromosomal cohesion have not been described.

Using an array of biochemical and cell biological methods, we mapped the amino acid interactive regions of the SA2 and Rad21 and found that SA1/2 physically interacts with Rad21 through a region from 301 to 750 aa. We also identified two SA-binding motifs of Rad21. Deletion or mutation of these two Rad21 domains (61–80 aa and 383–392 aa) disrupts the interaction of Rad21 and SA1/2, as well as some degree of cohesin dimerization that might be mediated through SA1/2. Importantly, the mutant Rad21 cannot efficiently rescue the premature sister chromatid separation caused by the knockdown of endogenous Rad21, indicating the structural and physiological importance of these two SA-binding motifs of human Rad21.

## Results

### The region 301–750 aa of the SA2 protein interacts with Rad21

To map the region(s) of SA2 that physically interacts with Rad21, we utilized the eukaryotic insect cell expression system to obtain recombinant human SA2 and Rad21 proteins. Baculoviruses overexpressing 6xHis-tagged SA2 and Flag-tagged Rad21 were generated. During the purification of SA2 from Sf21 cells, we noted that the full-length SA2 underwent partial degradation, resulting in a product of ∼120 kDa in size, the N-terminal 6xHis tag of which remained intact according to immunoblot (Figure S1A). An in-gel digestion by trypsin and chymotrypsin followed by mass spectrometry (MS) of the digested products indicated that the 120 kDa protein was comprised of the first 1122 residues of SA2 (Figure S1B). Based on these results, we conclude that the degradation of SA2 during purification occurred after amino acid residue T^1122^.

**Figure 1:**
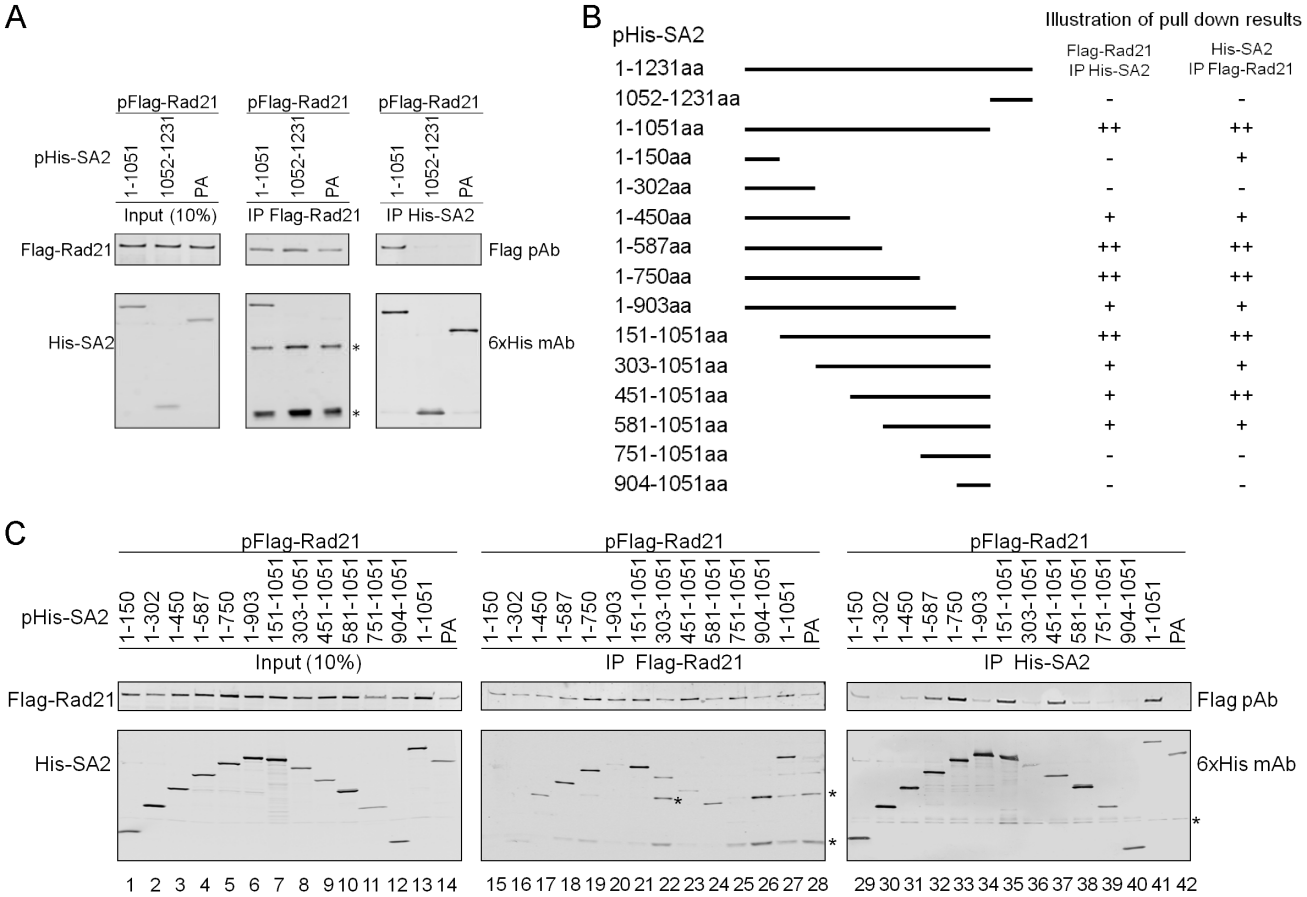
Characterization of the interaction between Rad21 and SA2 mutants. (**A**) Rad21 interacts with SA2 (1–1051 aa). Rad21 was co-expressed with either SA2 (1–1051 aa) or SA2 (1052–1231 aa) in Sf21 cells and co-purified by Ni-NTA or Flag beads. Rad21 co-expressed with the influenza A virus PA protein was used as a control. Western blot analysis was carried out using either the Flag polyclonal antibody (pAb) or the 6xHis monoclonal antibody (mAb). IgG bands are marked by asterisks (*). (**B**) Left panel shows the schematic illustration of the design of the SA2 deletion mutants used in (C). Right panel indicates the relative interaction strength of Rad21 and SA2 mutants in (C). ++: strong interaction; +: weak interaction; −: no interaction. (**C**) Flag-tagged Rad21 WT was co-expressed with His-tagged SA2 deletion mutants and co-purified by Ni-NTA or Flag beads. The influenza A virus PA was used as control. Nonspecific bands due to antibody cross-reaction are marked by asterisks (*).

A secondary structure prediction analysis using the program PredictProtein [23] showed an unstructured random coil region flanking T^1122^, which likely renders the expressed protein susceptible to proteolytic cleavage. Considering that the unstructured region may function as a domain linker, we generated two new baculoviruses, one expressing the first 1051 amino acid residues of SA2, with the entire unstructured coil region removed, and the other expressing the remainder of SA2 from residues 1052 to 1231, both containing a 6xHis tag at the N-terminus. Full-length Rad21 (1–631 aa), Flag-tagged at the N-terminus, was then expressed in Sf21 insect cells along with either SA2 (1–1051 aa) or SA2 (1052–1231 aa). Co-purification assays were performed using either Ni-NTA affinity beads to pull down the 6xHis-SA2 or the anti-Flag M2 affinity resin (agarose beads conjugated with Flag monoclonal antibody) to pull down Flag-Rad21. The samples were analyzed by Western blot (Figure 1A). Insect cells infected with Rad21 and the 6xHis-tagged PA protein of influenza A virus were used as a negative control for the co-purification assays. The reciprocal Ni-NTA and Flag co-purification results indicated that SA2 (1-1051 aa) but not the C-terminal SA2 (1052–1231 aa) can co-purify with Rad21, suggesting that the N-terminal 1051 aa are sufficient and the C-terminal 180 aa of SA2 are dispensable for the Rad21-SA2 interaction *in vitro* (Figure 1A).

To further narrow down the region of SA2 responsible for the Rad21-SA2 interaction, we generated baculoviruses overexpressing progressive SA2 deletion mutants with 150 amino acids increments/decrements from either the N- or the C-terminus of SA2 (1–1051 aa) (Figure 1B). As before, Flag-tagged Rad21 was expressed in Sf21 cells along with each of the 6xHis-tagged SA2 deletion mutants. Co-purifications were performed with Ni-NTA and Flag beads and analyzed by Western blot as described above. Co-purification results showed that the N-terminal (1–300 aa) and the C-terminal (751–1051 aa) regions of SA2 are not critical for interaction with Rad21 (Figure 1C). With the exception of SA2 (1–903 aa), other SA2 fragments containing the amino acid region of 301–750 interacted with Rad21 (Figure 1C, lanes 17–19, 21 & 32–33, 35–37). The interaction between Rad21 and SA2 (1–903 aa) was very weak (Figure 1C, lanes 20 & 34) and apparently was not due to less protein in the input sample (Figure 1C, lane 6). The interaction of Rad21 and SA2 (1–903 aa) might be reduced by a disturbed structure or protein misfolding. Indeed, a protein domain prediction using the program GlobPlot [24] showed that SA2 (850–940 aa) might form a globular domain, which supports our reasoning. Based on the information above, we conclude that the region of SA2 interacting with Rad21 is located within 301–750 aa region.

### SA2 interacts directly with the Rad21 central region in Sf21 insect cell system

**Figure 2:**
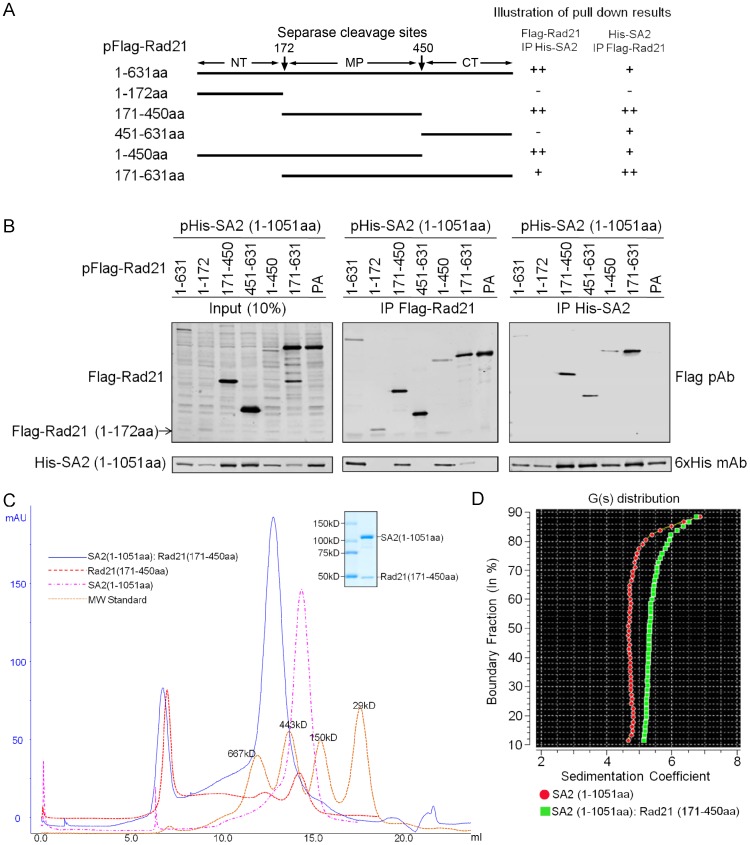
SA2 interacts with middle part of Rad21. (**A**) Schematic illustration of the Rad21 deletion constructs (left panel) and Rad21-SA2 interaction results from (B) (right panel). ++: strong interaction; +: weak interaction; −: no interaction. (**B**) Rad21 interacts with SA2 through the middle region. His-SA2 (1–1051 aa) was expressed along with Flag-Rad21 WT and deletion mutants and co-purified with Ni-NTA or Flag beads. SA2 (1–1051 aa) co-expressed with Flag tagged PA protein was used as a negative control. (**C**) Gel filtration chromatogram for the SA2 (1–1051 aa):Rad21 (171–450 aa) complex. SA2 (1–1051 aa) and Rad21 (171–450 aa) formed a stable complex. Inset shows the Coomassie-stained gel of the SA2:Rad21 complex purified by gel filtration. (**D**) Velocity sedimentation results for SA2 (1–1051 aa) and the SA2 (1–1051 aa): Rad21 (171–450 aa) complex. The complex shows an increase in the sedimentation coefficient compared to SA2 alone.

Previous studies have shown that yeast Scc3 directly interacts with Scc1 via the C-terminal Separase cleavage fragment [5]. In humans, Rad21 is cleaved by Separase at the onset of anaphase at R^172^ and R^450^ [16]. To determine the region of the human Rad21 that interacts with SA2, Rad21 deletion mutants were designed with reference to the two Separase cleavage sites and the corresponding baculoviruses were generated (Figure 2A). SA2 (1–1051 aa) was expressed in Sf21 cells along with Rad21 or its deletion mutants, and co-purification of the complex was performed to examine the interaction between SA2 and the Rad21 deletion mutants (Figure 2B). Insect cells infected with 6xHis-tagged SA2 (1–1051 aa) and Flag-tagged PA protein were used as a negative control for the co-purification assays. The results showed that SA2 (1–1051 aa) can form a complex with Rad21 (171–450 aa) but not with Rad21 (1–172 aa) or Rad21 (451–631 aa) (Figure 2B, middle panel). However, in the reverse co-purification, SA2 (1–1051 aa) does appear to form a complex with Rad21 (451–631 aa) (Figure 2B, right panel) but not with Rad21 (1–172 aa). The discrepancy may be due to a non-specific *in vitro* interaction between SA2 and Rad21 (451–631) in His-SA2 IP contributed by overexpressed recombinant Rad21 (451–631) protein.

### SA2 and Rad21 form a stable complex

To further investigate if the human SA2 directly binds to Rad21 and to validate the putative interacting domains of SA2 and Rad21 identified from the *in vitro* studies above, we co-expressed SA2 (1–1051 aa) and Rad21 (171–450 aa) in insect cells in which no other human cohesin proteins were present. The SA2 (1–1051 aa):Rad21 (171–450 aa) complex was purified using a Ni-NTA affinity column, followed by anion exchange and gel filtration (Figure 2C). The complex was eluted as a single peak in the gel filtration chromatography at a position earlier than SA2 (1–1051 aa) alone. Analytical ultracentrifugation was then performed for both the SA2 (1–1051 aa) and the SA2 (1–1051 aa):Rad21 (171–450 aa) complex. Velocity sedimentation data showed that the sedimentation coefficient of SA2 (1–1051 aa):Rad21 (171–450 aa) was indeed greater than that of SA2 (1–1051 aa) alone (Figure 2D), suggesting that SA2 (1–1051 aa):Rad21 (171–450 aa) can form a stable complex *in vitro*.

### 10 aa α-helix on middle part of Rad21 interacts with SA2

**Figure 3:**
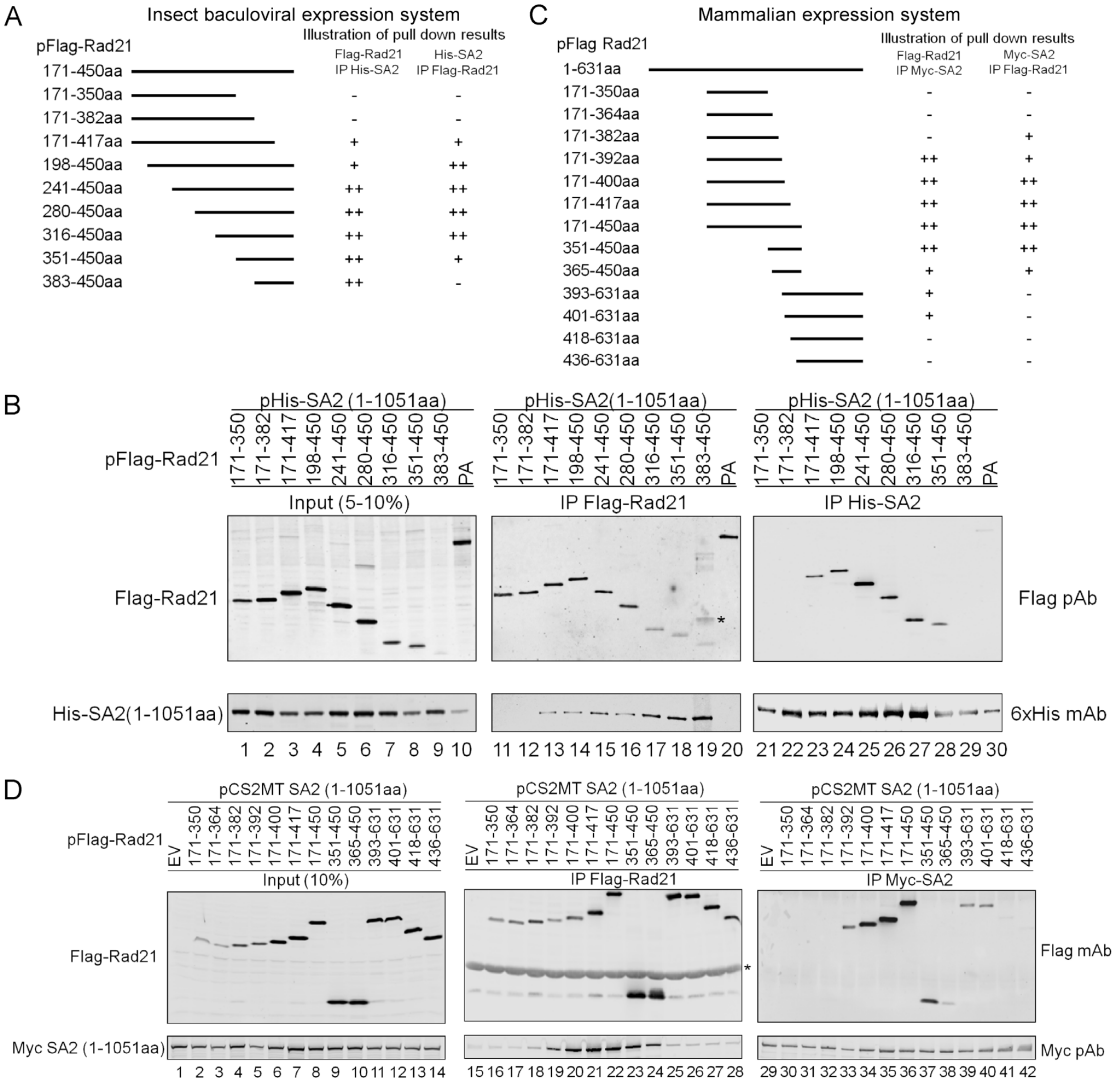
SA2 interacts with a 10 aa region of middle part Rad21. (**A**) Schematic illustration of the middle portion Rad21 deletion constructs made in the baculovirus system and interaction results of Rad21-SA2 from (B). ++: strong interaction; +: weak interaction; −: no interaction. (**B**) Rad21 (171–382 aa) does not interact with SA2. His-SA2 (1–1051 aa) was expressed along with the Flag tagged Rad21 deletion mutants and co-purified with Ni-NTA or Flag beads. Antibody cross-reaction bands are marked by asterisk (*). (**C**) Schematic illustrations of Rad21 deletion constructs in the context of the full length Rad21 in the mammalian expression vector pFlag CMV2 and interaction results from (D). ++: strong interaction; +: weak interaction; −: no interaction. (**D**) Rad21 383–392 aa region is critical for interacting with SA2. Myc-SA2 (1–1051 aa) was co-transfected along with the Flag-Rad21 deletion mutants and immunoprecipitated with Flag or Myc beads and probed with either the Myc polyclonal antibody (Myc pAb) or the FLAG mAb. Flag empty vector (EV) was used as a negative control.

To narrow down the polypeptide region of Rad21 that interacts with SA2, we utilized two approaches: 1) complex formation in the baculovirus expression system and 2) co-expression and immunoprecipitation (IP)-Western blot analysis in mammalian cells. Baculoviruses overexpressing progressive Rad21 deletion mutants with ∼35 amino acids increments/decrements from either the N- or C-terminus of the Rad21 middle region (171–450 aa) were generated (Figure 3A). SA2 (1–1051 aa) along with each of the Rad21 deletion mutants were expressed in Sf21 cells. The complex was purified using Ni-NTA or Flag beads and analyzed using Western blot. As shown in Figure 3B, Rad21 (351–450 aa), but not Rad21 (171–382 aa), could form a complex with SA2 (1–1051 aa) (Figure 3B, lanes 18 and 28 vs. lanes 12 and 22). While Flag-Rad21 (383–450 aa) co-purified His-SA2 (1–1051 aa) using Flag-beads (Figure 3B, lane 19), in the reciprocal IP, His-SA2 failed to co-purify with Rad21 (383–450 aa) (Figure 3B, lane 29), likely due to the low expression level of Flag-Rad21 (383–450 aa) (Figure 3B, lane 9). Collectively, the above results indicated that 351–450 aa of Rad21 is sufficient for its interaction with SA2, and Rad21 (383–450 aa) may be the minimal interacting region to form a Rad21-SA2 complex *in vitro* in this series of deletions using insect cells.

To further narrow down the interacting domain of Rad21 with SA2, we made another set of Rad21 constructs with deletion at the 383–450 region (Figure 3C) and validated the interaction of Rad21 and SA2 using a mammalian cell expression system. SA2(1–1051 aa) was cloned into the pCS2MT vector with a 6xMyc epitope at the N-terminus, and a series of Rad21 mutants were cloned into the pFlag CMV2 vector carrying a Flag epitope at the N-terminus (Figure 3C). SA2 (1–1051 aa) was transfected into 293 T cells along with each of the Rad21 deletion constructs. Co-IP with Flag beads and Myc monoclonal antibody (mAb)-conjugated agarose beads (Myc beads) were then used to analyze the interactions between SA2 and the Rad21 mutants (Figure 3D). Co-IP results showed that Rad21 (171–392 aa), but not the Rad21 (171–382 aa) or Rad21 (393–631 aa), were able to pull down SA2 (1–1051 aa) (Figure 3D, lane 19 vs 18 and 25; lane 33 vs 32), indicating that amino acids 383–392 are critical for the Rad21-SA2 interaction in human cells.

**Figure 4:**
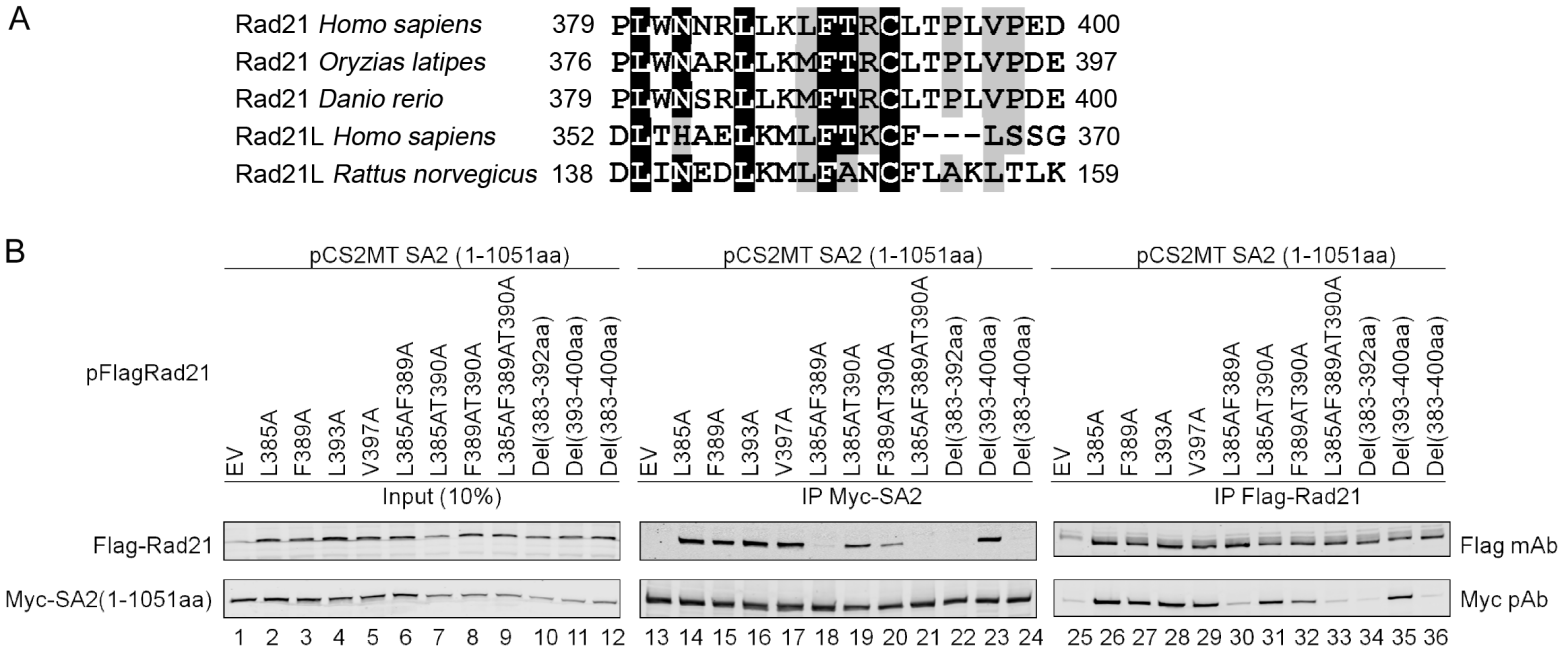
L^385^ and F^389^ are critical for Rad21 to interact with SA2. (**A**) Sequence alignment of Rad21 from various vertebrate species. The sequence alignment was prepared using BioEdit (http://www.mbio.ncsu.edu/bioedit/). The conserved Rad21 L^385^, F^389^ and T^390^ residues were used for making site-directed mutations. (**B**) Co-immunoprecipitation analysis indicating the importance of L^385^, F^389^, and T^390^ residues on Rad21 for its interaction with SA2. Flag-Rad21 and Myc-SA2(1–1051 aa) were expressed in 293 T cells. Co-IP was performed 48 h after transfection.

A secondary structure prediction for Rad21 (171–450 aa) using the program PredictProtein [23] revealed that the entire region is largely unstructured/disordered with only three α-helices, among which only two were predicted with high probability (Figure S2). Interestingly, one of these two highly probable α-helices is formed by 383–392 aa. An alignment of the Rad21 proteins from various vertebrate species indicated the presence of several conserved amino acid residues, including L^385^, F^389^, and T^390^ (Figure 4A). To confirm that 383–392 aa are critical for the Rad21-SA2 interaction and to identify the amino acids that are essential for this interaction, a Rad21 deletion mutant Del(383–392 aa) and several site-directed mutants were generated (Figure 4B). After Flag-Rad21 and Myc-SA2(1–1051 aa) were expressed in 293 T cells, co-IP results confirmed that the full-length Rad21 devoid of 383–392 aa failed to pull down SA2 (1–1051 aa) (Figure 4B, lanes 22 and 34). The mutation of two conserved amino acids L^385^ and F^389^ to alanine (A) within this 10 aa stretch in Rad21 significantly reduced its interaction with SA2 (Figure 4B, lanes 18 and 30). The combined mutations of all three conserved residues L^385^, F^389^, and T^390^ to alanine was able to abrogate the interaction between Rad21 and SA2 (Figure 4B, lanes 21 and 33), indicating that the three conserved amino acids L^385^, F^389^, and T^390^ are essential for the Rad21-SA2 interaction *ex vivo*. This result is consistent with an earlier report that the region 362–403 aa of Rad21 interacts with SA1 [22].

### Interaction of SA-binding deficient Rad21 mutants and other cohesin subunits

**Figure 5:**
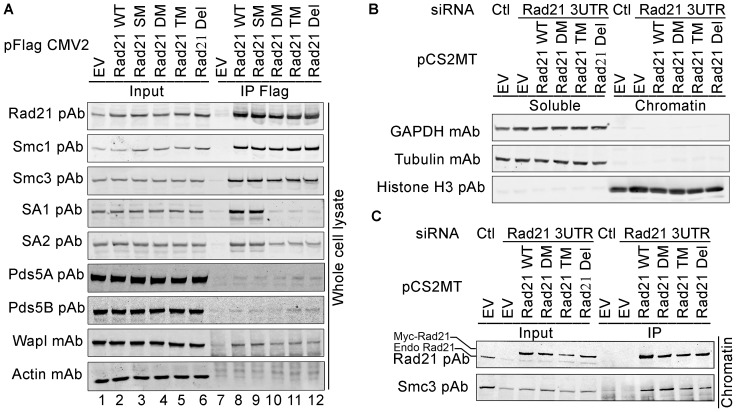
Co-immunoprecipitation of other cohesin subunits by Rad21 with mutations or deletion on MP SA-binding motif. (**A**) 293 T cells were transfected with pFlag CMV2 Rad21 WT or mutants and co-immunoprecipitation was performed using whole cell lysate. Cells transfected with empty vector (EV) was used as control. Immunoblotting shows the cohesin core subunits and associating proteins immunoprecipitated by Rad21 WT and mutants. (**B-C**) 293 T cells were transfected with pCS2MT Rad21 WT or mutants. EV was used as control. Endogenous Rad21 was knocked down with Rad21 3′-UTR siRNA. Scrabbled siRNA was used as control (Ctr). Proteins from chromatin fraction were isolated and used for IP. Immunoblotting shows the chromatin fraction was not contaminated by the soluble fraction (B) and like WT Rad21, mutant Rad21 was found on chromatin and co-immunoprecipitated by cohesin-Smc3 (C). EV: empty vector, WT: wild type, DM: L385A T390A, TM, L385A F389A T390A, Del: del(383–392 aa).

To investigate if SA2-binding deficient Rad21 mutants fail to co-immunoprecipitate endogenous SA2, Flag-Rad21 WT or mutants were expressed in 293 T cells and immunoprecipitated. Co-IP of endogenous cohesin subunits was analyzed using Western blot. In contrast to Rad21 WT and the single mutant (SM) L385A (Figure 5A lane 8–9), Rad21 double mutant (DM) L385A F389A, triple mutant (TM) L385A F389A T390A and deletion mutant [Del(383–392 aa)] significantly reduced the co-IP of endogenous SA1 and SA2 (Figure 5A, lane 10–12, Figure S3A). To further confirm that the interaction of SA1 and Rad21 is indeed disrupted by SA2-binding deficient Rad21 mutants, 293 T cells were co-transfected with Myc-SA1 and Flag-Rad21 constructs, and co-IP results verified that the Flag-Rad21 mutants significantly reduced the interaction with SA1 (Figure S4). Reduction of both SA1 and SA2 co-immunoprecipitation by Rad21 DM, TM, and Del mutants suggests that SA1 and SA2 bind to the same region of Rad21. Thus, we call Rad21 383–392 aa region as MP SA-binding motif because this region resides on the MP of Rad21 after Rad21 is cleaved by Separase at R172 and R450 (Figure 2A).

**Figure 6:**
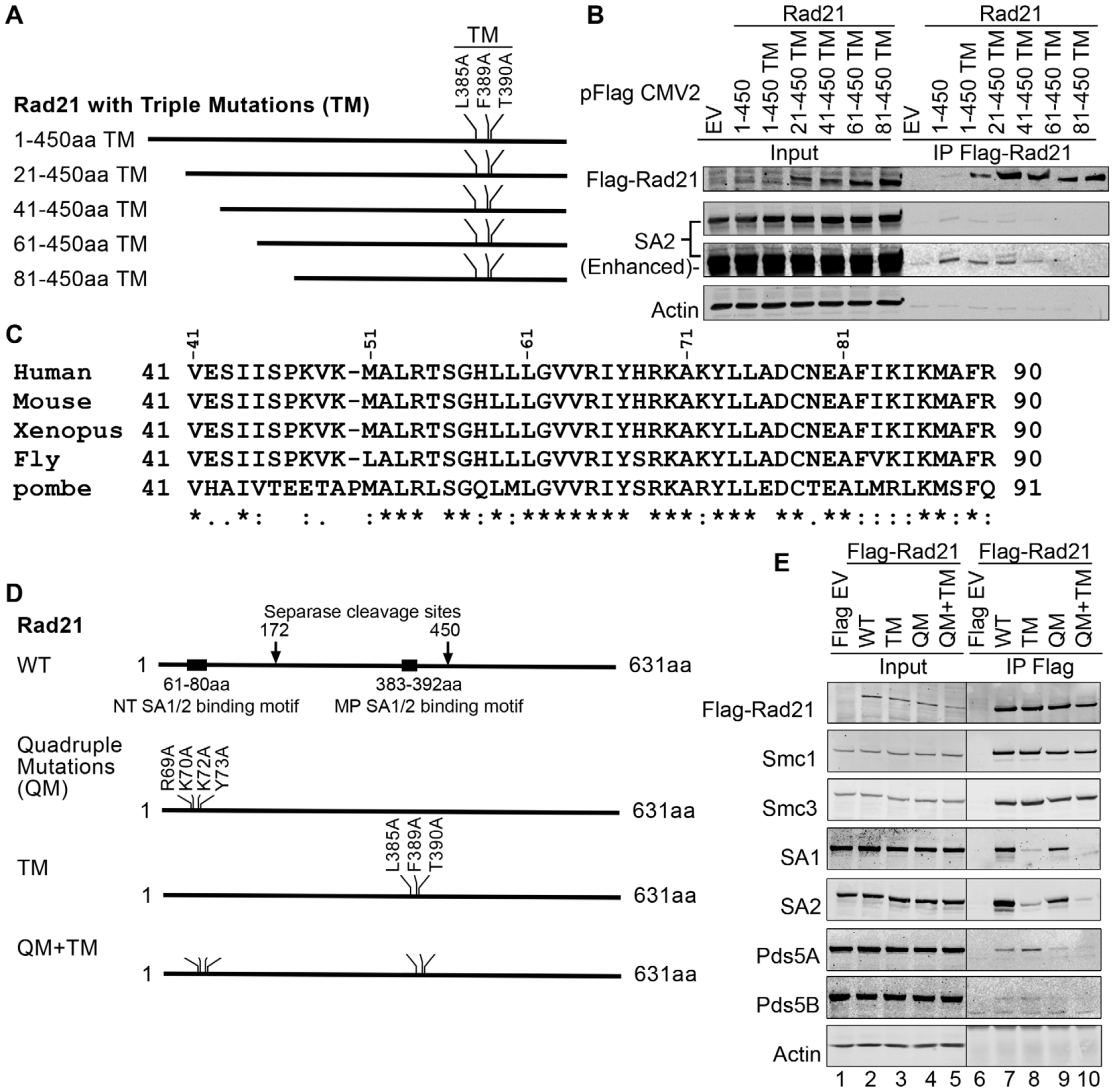
Identification of SA-binding site on N-terminus of Rad21. (**A**) Schematic illustration shows the N-terminal truncated mutants of Rad21 (1–450 aa) with triple mutations (TM) on middle part of SA-binding motif. The cDNA was cloned into pFlag CMV2 vector. (**B**) Immunoblotting of SA2 co-IP by Rad21 (1–450 aa) WT and mutants. 293 T cells were transfected with the constructs shown in (A) and IP was performed 40 h after transfection. Deleting the first 80 aa of Rad21 (1–450 aa) TM inhibits the co-IP of SA2. The second and third panels are from the same blot. The third panel was enhanced for better visualization. (**C**) Clustal format alignment of Rand21 (41–90 aa) by MAFFT L-INS-i (v7.015b). The bottom line shows the conserved amino acids from fission yeast (*S*. *pombe*) to human Rad21. Invariant, conserved, and semi-conserved residues are indicated by an asterisk (*), colon (:), and period (.), respectively. (**D**) Schematic illustration shows the full length Rad21 and the mutations on the two SA-binding motifs located on the NT and MP of Rad21, respectively. The cDNAs of Rad21 WT and mutants with quadruple mutations (QM) on the NT SA-binding motif and/or triple mutations (TM) on the MP SA-binding motif were cloned into pFlag CMV2 vector. (**E**) Immunoblotting of co-IP of cohesin subunits by Rad21 WT or mutants. The dividing lines indicate that interfering lanes have been spliced out.

To examine if the disruption of the Rad21-SA1/2 interaction has any effect on the association of Rad21 with other cohesin core subunits, the IP products pulled down by Flag-tagged Rad21 proteins from the above experiments were analyzed using antibodies against other cohesin core subunits and associating proteins. The immunoprecipitation of both Smc1 and Smc3 remained unchanged in all the Rad21 mutants compared to the WT control (Figure 5A, S3A), indicating that the disruption of the Rad21-SA2 interaction has no apparent effect on the Smc1-Smc3-Rad21 interaction. Co-IP of cohesin-associated factor Wapl was also not affected by the MP SA-binding deficient Rad21 mutations (Figure 5A). The effect of the MP SA-binding deficient Rad21 mutations on Pds5 association could not be clearly judged in this experiment because both Pds5A and Pds5B were not co-immunoprecipitated well (Figure 5A). However, Figure 6E (lane 7 vs. 8) demonstrated co-IP of Pds5 was not disrupted by the mutations on the MP SA-binding motif of Rad21.

To confirm the Rad21 mutants can be incorporated into the cohesin complex and associated with chromosomes, we knocked down the endogenous Rad21 with siRNA against 3′-UTR of Rad21, expressed WT or mutant Rad21 in 293 T cells, and performed IP from chromatin fractions. The chromatin fraction without the contamination of soluble fraction was validated with GAPDH and tubulin monoclonal antibodies (mAb) (Figure 5B). The immunoprecipitation of Smc3 by all the Rad21 mutants in chromatin fractions was not affected (Figure 5C). Because Rad21 binding to Smc1 is required for its binding to Smc3 [25], the results confirmed that the Smc1-Smc3-Rad21 complex is not only present in soluble fraction, but it is also capable of binding to chromatin.

### N-terminus of Rad21 contains a second SA-binding site

We noticed that mutation or deletion of the SA-binding motif at 383–392 aa of Rad21 could not completely abrogate the interaction of SA1 and SA2 in the co-IP experiments (Figure 5A, S3A). We reasoned that there may be additional SA-binding site/s on Rad21. Using a set of pilot experiments in 293 T cells, we found that SA1 and SA2 could be co-immunoprecipitated by the N-terminal fragment (1–172 aa) of Rad21 (Rad21 NT) and the middle part (173–450 aa) of Rad21 (Rad21 MP), but not the C-terminal fragment (451–631 aa) of Rad21 (Rad21 CT) (Figure S5). As described above, we have identified 383–392 aa as one of the SA-binding motifs on Rad21 MP, and because there is no interaction of SA1/2 within the C-terminal (451–631 aa) domain of Rad21, we focused our analysis for a possible SA-binding motif on the N-terminal domain of Rad21 (Rad21 NT). We made deletion mutants with 20 aa increments from the N-terminus of Rad21 (1–450 aa), with triple mutations (TM) of L385A-F389A-T390A on the identified MP SA-binding motif (Figure 6A). The Rad21 proteins were expressed in 293 T cells, and IP was performed. The immunoblotting results showed that deletion of the first 40 aa of Rad21 (41–450 aa) reduced the co-IP of SA2, and deletion of first 80 aa (Rad21 81–450) completely abolished the co-IP of SA2 (Figure 6B). Because these Rad21 constructs contain triple mutations at L385F389T390 along with the deletions of C-terminus (451–631 aa) and a portion of the N-terminus of Rad21, the interaction with SA2 in the co-IP experiments was significantly decreased. The above data suggest that another SA2 interacting motif is located within the first 80 aa of the Rad21 NT.

First 150 aa of the N-terminus of Rad21 are highly conserved across different species (Figure 6C). By examining the amino acid sequence around 60 aa, we speculated that the region of 61–80 aa of Rad21 may be critical for SA1/2 interaction. Deletion of 61–80 aa region of Rad21 (1–450 aa) TM indeed blocked the co-IP of SA1/2 (data not shown). To further confirm these two SA-binding motifs (61–80 aa, 382–392 aa) in full-length Rad21, we made three full-length constructs with quadruple mutations (QM) of R69A, K70A, K72A and Y73A at the region of 61–80 aa and TM of L385A, F389A and T390A at the region of 383–392 aa either alone or in combination (Figure 6D). Rad21 mutants were expressed in 293 T cells and immunoprecipitated with Flag mAb conjugated agarose beads. Immunoblotting results demonstrated that the efficiency of co-immunoprecipitation of SA1/2 by Rad21 constructs is as follows: Rad21 WT<Rad21 QM< Rad21 TM, and Rad21 QM+TM failed to pull down SA1/2 (Figure 6E). Similar to the TM, the QM did not affect the co-IP of Smc1 and Smc3. However, unlike the Rad21 WT or the Rad21 TM, the Rad21 QM reduced the co-IP of Pds5 (Figure 6E). Based on the above data, we conclude that at least two SA-binding sites are on Rad21: one is located on the N-terminus, and the other is on the MP of Rad21 molecule. Similar to the MP SA-binding motif, we name the SA-binding domain on the N-terminus of Rad21 the NT SA-binding motif.

### Mutations on SA-binding motifs of Rad21 reduce Rad21-Rad21 dimerization and result in premature separation of sister chromatids

**Figure 7:**
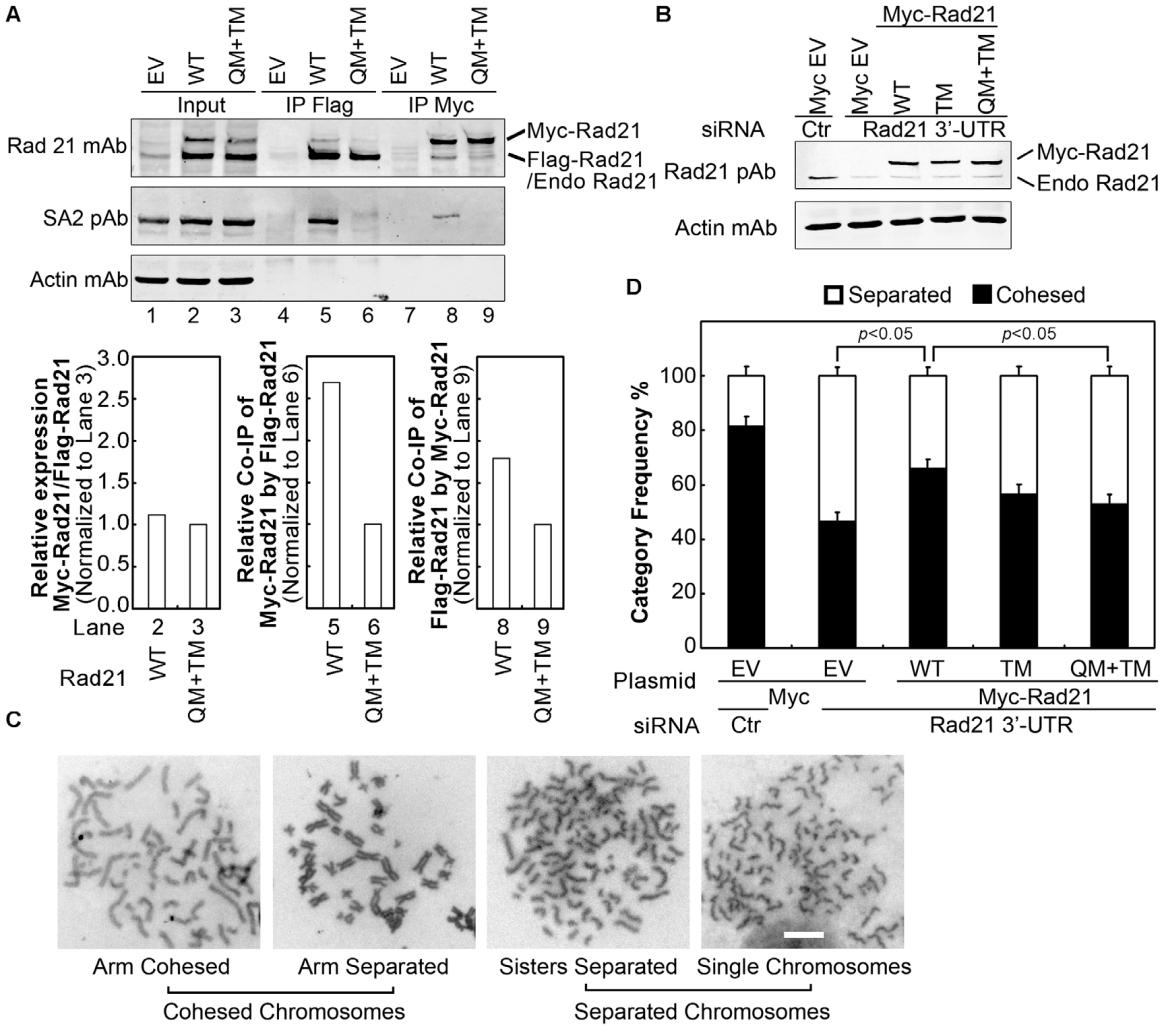
Reduced Rad21-Rad21 dimerization and defect in sister chromatid cohesion in cells expressing Rad21 with mutations on SA-binding motifs. (A) Co-IP of differentially tagged Rad21 with mutations on both NT and MP SA-binding motifs. Rad21 WT or mutants of QM+TM (see Figure 6D) were tagged with Flag or Myc on its N-terminus and expressed in 293 T cells. IP was performed with Flag mAb or Myc pAb conjugated agarose beads and immunoblotting was carried out. The bar graphs show the relative expression or co-IP level of Myc-Rad21 and Flag-Rad21. Rad21 WT bands were normalized using respective Rad21 QM+TM mutant bands. (B-D) Rad21 mutants cannot rescue the premature separation of sister chromatids caused by Rad21 knockdown. 293 T cells were treated with Rad21 3′-UTR siRNA or a control (Ctr) siRNA for 24 h before Myc-Rad21 WT or mutant plasmids was transfected. The knockdown of endogenous Rad21 and the expression of Myc-Rad21 were analyzed by Western blot using Rad21 pAb (B). Metaphase chromosome spread was performed and the status of sister chromatid cohesion was analyzed according to the categories shown in (C). About 100 mitotic cells were counted for each treatment. The frequency of mitotic cells was calculated and plotted in (D). EV, empty vector; WT, wild Type; QM, quadruple mutant R69A K70A K72A Y73A; TM, triple mutant L385A F389A T390A. Bar size = 10 µm. Values were compared using Student's *t*-test.

Previously, we proposed the handcuff model of cohesin configuration, in which we suggested the anti-parallel dimerization of Rad21 molecules through SA1/2. However, we did not rule out possible roles of other cohesin factors in the Rad21-Rad21 dimerization [17]. To test if mutations on SA-binding motifs of Rad21 disrupt Rad21-Rad21 dimerization, we performed co-IP of Flag and Myc epitope-tagged Rad21 molecules with SA-binding motif mutants. Immunoblotting results showed that mutation or deletion of the SA-binding motif at MP of Rad21 alone did not significantly reduce the Rad21-Rad21 interaction (Figure S3B). Myc-tagged Rad21 with mutation or deletion of the MP SA-binding motif could also co-immunoprecipitate endogenous Rad21 (Figure S3A). Similar results were observed with mutation or deletion of the SA-binding motif at N-terminus of Rad21 alone (data not shown). However, compared to wild type Rad21, the co-IP of Flag-Rad21 and Myc-Rad21 with mutations on both NT and MP SA-binding motifs (QM+TM) of Rad21 was significantly reduced (Figure 7A, lanes 5 & 6, 8 & 9), suggesting that SA1/2 links Rad21 proteins at multiple sites and also play an important role in forming the two-ring cohesin complex.

To examine the physiological consequence on the chromosomal cohesion after the interaction of SA1/2 and Rad21 is disrupted, Myc-tagged WT and mutant Rad21 were expressed in 293 T cells. Myc empty vector (EV) was used as a control. To examine the effect of the ectopic protein, endogenous Rad21 was knocked down using Rad21 3′-UTR siRNA. Compared to the samples treated with control siRNA, at least 80% of endogenous Rad21 protein in samples treated with Rad21 3′-UTR siRNA was knocked down (Figure 7B). The expression of WT and mutant Myc-tagged Rad21 was equivalent (Figure 7B). Metaphase chromosome spread analysis was performed, and mitotic cells from each sample were counted according to their separation status: 1) cohesed chromosomes that include chromatids linked at the centromeres with arm cohesed or separated and 2) separated chromosomes that include chromatids separated but still in alignment with each other and single chromatids randomly scattered (Figure 7C). Approximately 20% of the control siRNA treated cells showed separated chromatids. In comparison to the control siRNA treated cells, approximately 55% precocious sister chromatid separation was noted in the cells treated with Myc EV control plus Rad21 3′-UTR siRNA, the phenotype of which could be partially but significantly (p<0.05) rescued (∼20%) by overexpressing the ectopic WT Rad21 (Figure 7D). However, Rad21 construct with mutations on both the NT and MP SA-binding motifs (QM+TM) failed to significantly rescue the PCS phenotype caused by the Rad21 knockdown (Figure 7D). These results suggest that both the NT and MP SA-binding motifs of Rad21 are not only required for Rad21-SA2 interaction but also are physiologically important for sister chromatid cohesion.

## Discussion

It has been reported that SA2 associates with the tripartite cohesin ring through binding to Rad21 [5]. However, details of the Rad21-SA2 interaction at the molecular level and its consequence in chromosomal segregation are lacking. Here we show that the 301–750 aa region of SA2 interacts with Rad21. We have also identified two SA-binding motifs encompassing 61–80 aa and 382–292 aa regions of Rrad21. Deletion or mutations of these Rad21 motifs result in the failure of Rad21-Rad21 dimerization and precocious chromatid separation.

### SA1 and SA2 interact with Rad21 in a similar way

Using the insect cell expression system, we identified a broad region (301–750aa) of SA2 that interacts with Rad21. It is possible that a different portion of SA2 interacts with a different region of Rad21, which is evident from our studies in mammalian cells in which Rad21 contains at least two SA-binding motifs. In metazoa, phosphorylation of SA2 by Polo-like kinase 1 (Plk1) at the chromosome arms during prophase is required for the dissolution of arm cohesion [26]–[28]. The C-terminal SA2 region (residues 1052–1231aa) contains 12 of the 14 SA2 phosphorylation sites, including the seven highly phosphorylated sites [29]. An interesting note is that this C-terminal SA2 region is not required for SA2 binding to the SA-binding motif of Rad21, suggesting its regulatory function.

SA1, the other Scc3 ortholog in humans, contains 1258 amino acid residues and shares approximately 70% sequence identity with SA2. Most of the amino acid variations between SA1 and SA2 are in the 1–68 aa and 1075–1162 aa regions. SA1 shares 77% sequence identity with SA2 at the 301–751 aa region of SA2 that interacts with Rad21. The 301–751 aa region in SA2 also contains stromalin conserved domain (SCD), which is conserved in SA1, SA2, and SA3 [30], suggesting that similar molecular interactions may exist between SA1 and Rad21. Our results from mammalian cells show that the Rad21 mutants with reduced interaction with SA2 also decrease the interaction with SA1, indicating SA1 and SA2 interact physically with Rad21 in a similar way. The physiological significance of the 301–751 aa region in SA2 is also demonstrated by the recent findings of truncated and missense mutations in this region in a number of human cancers [20].

### Rad21 has two SA-binding motifs

SA1/2 is known to interact with cohesin via Rad21, but which portion of Rad21 binds to SA1/2 is not clear. According to the two Separase cleavage sites on human Rad21, we made three Rad21 constructs containing the NT, MP, and CT fragment, respectively, to map which part of Rad21 interacting with SA1/2. Our results show that in the insect cell expression system, only the MP of Rad21 can interact with SA2, whereas in mammalian cells both the NT and the MP fragments of Rad21 can co-immunoprecipitate SA2. However, the Rad21 CT cannot interact with SA1/2 in either system. Further studies indicate that the interaction between the MP of Rad21 and SA2 is restricted to a 10 aa α-helical region. In this 10 aa α-helical region, L^385^ and F^389^ are next to each other in the helix (Figure S6) and Rad21-SA1/2 interaction is disrupted when L^385^ and F^389^ are mutated into alanine, suggesting that this particular side of the α-helix interacts with SA1/2.

The Rad21 NT can co-immunoprecipitate endogenous Rad21 in 293 T and HeLa cells, but it fails to interact with SA2 in the insect cell expression system. Two possibilities explain this disparity. One is that SA1/2 interaction with Rad21 NT requires additional factors that mammalian cells, but not insect, cells have. One or more of cohesin-associated proteins, such as Pds5 and Sororin, are probable candidates to fulfill this function. The other possibility is that the C-terminus of SA2 is required for the interaction between Rad21 NT and SA2. The latter is ruled out because the C-terminus truncated SA2 has similar efficiency in co-immunoprecipitating Rad21.

The Rad21 MP but not the Rad21 CT interacting with SA1 and SA2 appears to be contradictory to the findings in yeast, where Scc1/Mcd1 NT Separase-cleavage fragment cannot co-purify Scc3, whereas the Scc1/Mcd1 CT Separase-cleavage fragment can [5]. Both the yeast Scc1/Mcd1 (566 aa) and the human Rad21 (631 aa) have two Separase cleavage sites. The cleavage sites of the yeast Scc1/Mcd1 are at R^180^ and R^268^, respectively [31], [32]. The sizes of the Separase-cleavage fragments of Scc1/Mcd1 NT (1–180 aa) and Rad21 NT (1–172 aa) are similar, but the Separase-cleavage fragments of yeast Scc1/Mcd1 CT (269–566 aa) and human Rad21 CT (450–631 aa) are remarkably different. Although Rad21 CT (451–631 aa) cannot immunoprecipitate SA1/2, its N-terminal extended version of Rad21 (254–631 aa) can (data not shown). It is possible that the co-purification of yeast Scc3 and Scc1/Mcd1 CT is dependent of its long MP, just like the Rad21 MP.

### Mutations of SA-binding motifs on Rad21 lead to defect of sister chromatid cohesion

According to the handcuff model, Smc1, Smc3, and Rad21 form a ring, and two of such rings are dimerized via the Rad21 molecules bridged by SA1/SA2 and potentially other cohesin-associated proteins [17], [18]. Therefore, Rad21-Rad21 interaction with mutations on MP SA-binding motif appears contradictory to the previous observation that knockdown of SA1/2 reduced the Rad21 dimerization [17]. However, when both the NT and MP of SA-binding motifs were mutated, the interaction between mutant Rad21 proteins was considerably reduced, which is consistent with our previous studies that suggest SA1/2 functions in bridging the two cohesin rings [17]. We also suggested earlier [17], [18] that, besides SA1/2, other cohesin-associated proteins may be involved in the bridging of two cohesin rings. Pds5A and Pds5B are the most likely candidates because they interact with SA1/2 [33], [34]. Pds5A/B can be co-immunoprecipitated by Rad21, which is not affected by the mutation or deletion of MP SA-binding motif (Figure 5A, 6E). However, Rad21-Pds5 interaction is abolished by the mutation of the NT SA-binding motif (Figure 6E), suggesting a tripartite interaction among Rad21, SA1/2, and Pds5A/B. Whether SA1/2 directly interacts with the NT SA-binding motif of Rad21 or requires additional interactions with Pds5A/B remains to be determined. Sororin is another potential candidate, which needs to be further investigated.

Depletion of endogenous Rad21 causes premature chromosomal separation, which can be rescued by ectopic expression of WT Rad21, but not significantly by Rad21 with mutations on both the SA-binding motifs. It is possible that the mutations on SA-binding motifs of Rad21 proteins hinder the two cohesin rings to form handcuff configuration, resulting in the inability to establish or maintain sister chromatid cohesion. How SA1/2 and cohesin-associated proteins cooperatively participate in linking the two cohesin rings would be of interest for future investigation.

## Materials and Methods

### Antibodies

Antibodies used in this study are listed below. Mouse monoclonal antibodies: Flag (Sigma-Aldrich, St. Louis, MO), Myc (Calbiochem, San Diego, CA.), His (BD Biosciences, San Jose, CA), Rad21 [35], SA2 mAb (Santa Cruz Biotech, Santa Cruz, CA), and Actin (Sigma-Aldrich). Rabbit polyclonal antibodies: Flag (Sigma-Aldrich), Myc (Sigma-Aldrich), Smc3 (Bethyl Laboratories, Inc., Montgomery, TX), and Rad21 pAb [35]. Goat polyclonal antibodies: SA1 and SA2 (Novus Biologicals, Littleton, CO), and Smc1α (Santa Cruz Biotech). All secondary antibodies were from Rockland Immunochemicals (Gilbertsville, PA).

### Cell culture

Sf21 cells (Invitrogen, Carlsbad, CA) were grown in Grace's media (Sigma-Aldrich) supplemented with 10% FBS at 27°C on a shaker at 110 rpm. 293 T cells (ATCC, Manassas, VA) were grown in DMEM supplemented with 10% FBS in a 37°C incubator under 95% humidity and 5% CO_2_.

### Cloning of SA2 and Rad21 deletion mutants in baculovirus system

SA2 or Rad21 cDNA was cloned into the pFastBac1 vector (Invitrogen). Recombinant viruses were generated according to the Bac-to-Bac Baculovirus Expression System protocol (version D) (Invitrogen).

### Cloning of SA2 and Rad21 deletion mutants in mammalian cells

SA2 (1–1051 aa) was cloned into the pCS2MT vector, and Rad21 deletion mutants were cloned into pFlag CMV2, pCS2MT, or pCruz HA vectors.

### Transfection

Plasmids were transfected into 293 T cells using the calcium phosphate method [36]. The medium was changed 16 h after transfection.

### In-gel digestion and Mass Spectrometry (MS)

The SA2 sample was electrophoresed on a SDS-PAGE gel and stained with Coomassie staining solution (Bio-Rad, Berkeley, CA). The band containing the SA2 degradation product was cut out and sent to the Tufts University Core Facility for in-gel digestion and MS analysis.

### Co-purification of baculovirus expressed proteins

Ten million Sf21 cells were harvested 48 h post-infection and lysed in 1 ml lysis buffer (50 mM Tris-HCl pH 7.4, 50 mM NaCl, 1% Triton X-100, and 1 mM PMSF). For Ni-NTA co-purification, cell extracts were supplemented to contain 20 mM imidazole and 300 mM NaCl before being incubated with 30 µl Ni-NTA resin (Qiagen, Valencia, CA) at 4°C for 1 h. The resin was washed three times with 1 ml Ni-NTA buffer A (50 mM Tris-HCl pH 7.5, 300 mM NaCl, and 20 mM imidazole), and the tagged protein was eluted with 2× SDS sample buffer (125 mM Tris-HCl, pH 6.8, 20% glycerol, 4% SDS, 0.1% bromophenol blue, and 100 mM DTT). For Flag beads co-purification, the cell extracts was adjusted to contain 150 mM NaCl and incubated with 20 µl Flag beads at 4°C for 3 h. The beads were then washed three times with 1 ml PBS buffer (137 mM NaCl, 2.7 mM KCl, 8.1 mM Na2HPO4, and 1.5 mM KH2PO4, pH 7.4), and the tagged protein was eluted with 2× SDS sample buffer.

### Purification of the SA2:Rad21 complex

Two liters of Sf21 cells were co-infected with baculovirus overexpressing the 6xHis tagged SA2 (1–1051 aa) and Flag tagged Rad21 (171–450 aa) at a multiplicity of infection of 4∶4. Sf21 cells were harvested 60 h post-infection. Cell pellets were then suspended in Ni-NTA buffer A (50 mM Tris-HCl pH 7.5, 300 mM NaCl, and 20 mM imidazole). Following lysis and centrifugation, the supernatant was loaded onto Ni-NTA beads (Qiagen). The mixture was gently stirred at 4°C for 1 h, followed by an 8 min centrifugation at 2,000 xg to separate the beads from the flow through. The beads were then washed by a washing buffer containing 20 mM imidazole. After washing, the complex was eluted by an elution buffer containing 250 mM imidazole. The eluted protein solution containing the complex was then diluted three times with FPLC buffer A (50 mM Tris-HCl pH 7.5, 1 mM EDTA, 1 mM NaN_3_, and 10% glycerol) and loaded onto a 5 ml Hitrap Q column (GE Healthcare, Pittsburgh, PA), which is an anion exchange column. Samples were eluted using an increasing gradient of salt at pH 7.5. Fractions containing the complex were collected, concentrated, and loaded onto a 24 ml Superose 6 gel filtration column (GE Healthcare). A gel filtration buffer (25 mM Tris-HCl pH 7.5, 200 mM NaCl, and 5% glycerol) was used to elute the protein.

### Immunoprecipitation and Western blotting

Protein isolation and immunoprecipitation were performed as reported previously [17]. Cell lysates or IP samples were electrophoresed on 5%–20% gradient SDS gels and transferred to nitrocellulose membranes (Bio-Rad). The membranes were blotted with blocking buffer (LI-COR Biosciences, Lincoln, NE) for 1 h and probed with primary antibody for 1 h, after which the membranes were washed three times with TBST buffer (20 mM Tris-HCl pH 7.4, 150 m NaCl, and 0.1% Tween 20). The membranes were then probed with appropriate secondary antibodies labeled with IRDye 800 or Cy 5.5 for 1 h, followed by three washes with TBST buffer. The membranes were then visualized by an Odyssey infrared scanner (LI-COR Biosciences).

### Preparation of chromatin fraction from 293 T cells

293T cells were lysed, and pellets were collected using centrifugation. The pellets were then suspended in cell lysis buffer containing 1mM MNase. After sonication, the samples were incubated in 37°C water bath for 10min. The supernatants were collected as the chromatin fraction after centrifugation at 20000xg for 15min.

### Analytical ultracentrifugation and data analysis

Sedimentation velocity experiments were performed using a Beckman Coulter Optima XL-A analytical ultracentrifuge. The optical density (OD) values of the samples were obtained against a water reference at 230nm. The sedimentation velocity runs were performed at 30,000rpm (AN60 Rotor) at 4°C for 10h. One hundred scans were recorded for each sample at a radial step size of 30 µm. Data analysis was performed with UltraScan version 9.9 (Ultrascan [37], http: //www.ultrascan.uthscsa.edu). Time invariant noise and radially invariant noise were subtracted from the sedimentation velocity data by 2-dimentional spectrum analysis [38]. G(s) sedimentation coefficient distributions were obtained with the enhanced van Holde-Weischet analysis [39].

### SiRNA and transfection

Silencer Negative Control siRNA (Applied Biosystems, Foster City, CA) was used as a negative control. Rad21 siRNA (Qiagen) was transfected into cells using DharmaFECT™1 (Dharmacon, Lafayette, CO).

### Metaphase chromosome spread

Cells were treated by trypsin, after which they were centrifuged at 800 g for 6 min. 10 ml of a pre-warmed hypotonic solution (0.075 M KCl) was added to gently agitate the pellets, followed by incubation at 37°C for 15 min to break the membrane. One ml of fresh fixative (methanol: glacier acetic acid = 3∶1) was then added before the cells were centrifuged at 800 g for 6 min and the supernatants were discarded. Four ml of fixative was added to each sample before they were incubated at room temperature for 30 min. Fixative was changed twice and cells were re-suspended in 200 µl fixative after the final centrifugation. To create slides, 30 µl mixture was taken from each sample and dropped on angled slides from 10 inches above. The slides were then air-dried and stained in Giemsa solution for 10 min before being rinsed with water ten times. About 100 metaphase cells were counted for each treatment using a Zeiss AxioSkop 40 microscope (Carl Zeiss, Thornwood, NY).

### Data analysis

All the experiments described in this study were performed multiple times (2 or more times) and the representative figures are shown. The results of premature separation of chromatids were analyzed using Student's *t*-test.

## Supporting Information

Figure S1 Identification of the SA2 degradation product. (**A**) Western blot of purified SA2 full length protein. The 6xHis tag at the N-terminal of full length SA2 was detected by 6xHis mAb. There is a ∼120 kDa degradation product also containing the N-terminal tag. (**B**) In-gel digestion and peptide identification by HPLC/MS. T^1122^ is the last amino acid identified by MS. The peptide coverage at the N-terminal region is not shown. The lines below the amino acid sequence indicate the peptides were identified by mass spectrometry.
(TIF)

Figure S2 Secondary structure prediction for Rad21 (171–450 aa). Rad21 amino acid sequence is colored based on residue types (e.g. blue for positively charged, red for negatively charged, green for hydrophobic, silver for polar, etc). PROF_sec predicts the secondary structure (H = Helix). Rel_Sec shows the reliability index of the PROF_sec prediction (0 = low, 9 = high).
(TIF)

Figure S3 Mutations on middle part SA1/2-binding motif of Rad21 do not affect Rad21-Rad21 interact and co-immunoprecipitate Smc1/3. 293 T cells were transfected with pCM2 MT Rad21 (and pFlag CMV2 Rad21 for (B)). Empty vector was used as control. Co-immunoprecipitation was performed using whole cell lysate. (**A**) Immunoblotting shows the cohesin core subunits including endogenous Rad21 were co- immunoprecipitated by Myc-Rad21 WT and mutants. (**B**) Immunoblotting of the co-IP of Flag-Rad21 and Myc-Rad21, which does not affect by the mutation on the middle part of SA1/2 binding motif of Rad21.
(TIF)

Figure S4 Immunoblotting shows co-immunoprecipitation of Myc-SA1 and Flag-Rad21 WT and mutants. 293 T cells were transfected with pCS2 MT SA1 and pFlag CMV2 Rad21 WT or mutant with mutations on middle part of SA1/2-binding motif. Co-immunoprecipitation was performed using whole cell lysate. EV: empty vector; WT: wild type; SM: L385A; DM: L385A T390A; TM: L385A F389A T390A; Del: del(383–392 aa).
(TIF)

Figure S5 N-terminal Rad21 (1–172 aa) and middle part of Rad21 (173–450 aa) contains SA1/2-binding motif. 293 T cells were transfected with the appropriate plasmids as shown. IP was performed using cell lysates 40 h after transfection. (**A**) Schematic illustration shows the Rad21 truncated mutants. The Separase cleavage sites at 172 and 450 (arrows) and SA1/2-binding motif at 383–392 aa (rectangle block) are shown. WT: wild type; NT: N-terminus; MP: middle part; CT: C-terminus. (**B**) Rad21 NT co-immunoprecipitates itself as well as SA1, SA2 and endogenous Rad21 (lane 5), but not Smc1 and Smc3. (**C**) Flag- and HA-Rad21 MP co-immunoprecipitate each other as well as SA1 and SA2, but fail to co-immunoprecipitate Smc1, Smc3 and Rad21 (lane 4). (**D**) Flag- and HA-Rad21 CT co-immunoprecipitate Smc1 and Smc3, but fail to co-IP each other and SA1/2 (lane 4).
(TIF)

Figure S6 Helical wheel illustration of the Rad21 383–392 aa. L^385^ and F^389^ are next to each other in the helix. The helical wheel was created using the following website: http://rzlab.ucr.edu/scripts/wheel/wheel.cgi?sequence=ABCDEFGHIJLKMNOP&submit=Submit (Zidovetzki, R., Rost, B., Armstrong, D. L., and Pecht, I. (2003) *Biophys. Chem.* **100**, 555–575).
(TIF)
