# Correction: Characterization of the Interaction between the Cohesin Subunits Rad21 and SA1/2

**DOI:** 10.1371/annotation/69ab23f6-7d99-4098-85ca-999da9093ec8

**Published:** 2013-08-06

**Authors:** Nenggang Zhang, Yunyun Jiang, Qilong Mao, Borries Demeler, Yizhi Jane Tao, Debananda Pati

A second grant given by the National Cancer Institute was incorrectly omitted from the Funding statement. The number of the second grant is: 1RO1 CA109478. 

