# Supplementary figures and images for: Characterization of the Interaction between the Cohesin Subunits Rad21 and SA1/2
Source: PLoS One. 2013 Jul 12;8(7):e69458. doi: 10.1371/journal.pone.0069458 (PMC3709894; doi:10.1371/journal.pone.0069458)

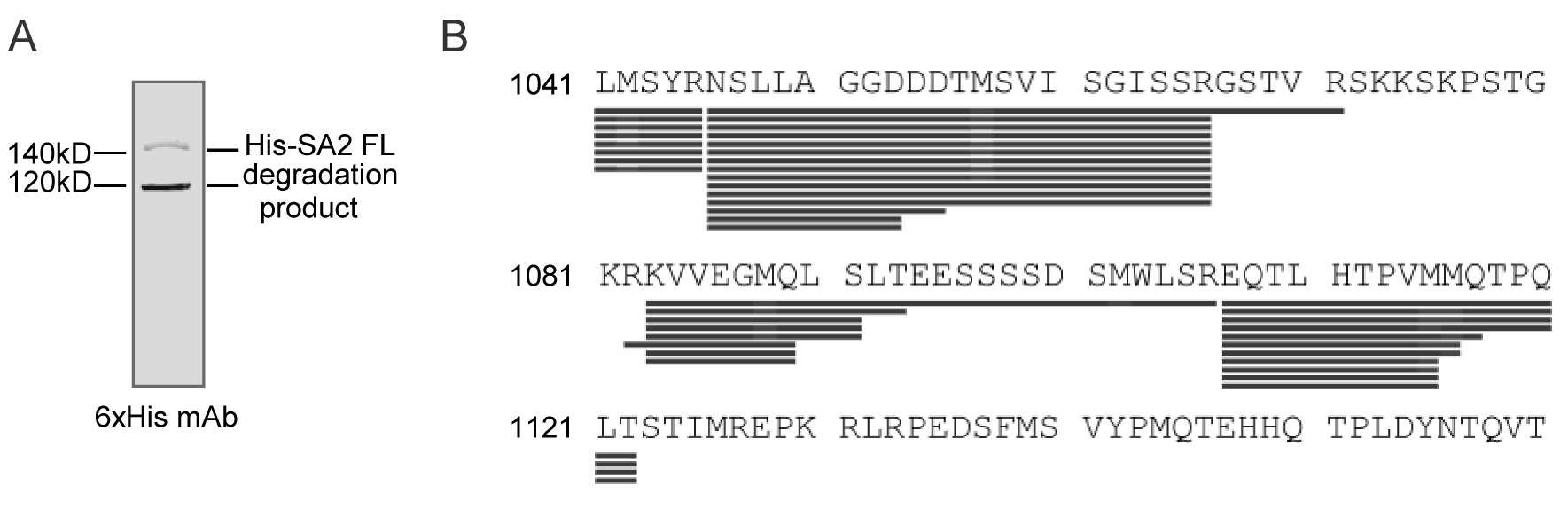

Supplement: Figure S1 — Identification of the SA2 degradation product. (A) Western blot of purified SA2 full length protein. The 6xHis tag at the N-terminal of full length SA2 was detected by 6xHis mAb. There is a ∼120 kDa degradation product also containing the N-terminal tag. (B) In-gel digestion and peptide identification by HPLC/MS. T1122 is the last amino acid identified by MS. The peptide coverage at the N-terminal region is not shown. The lines below the amino acid sequence indicate the peptides were identified by mass spectrometry. (TIF) [file pone.0069458.s001.tif]

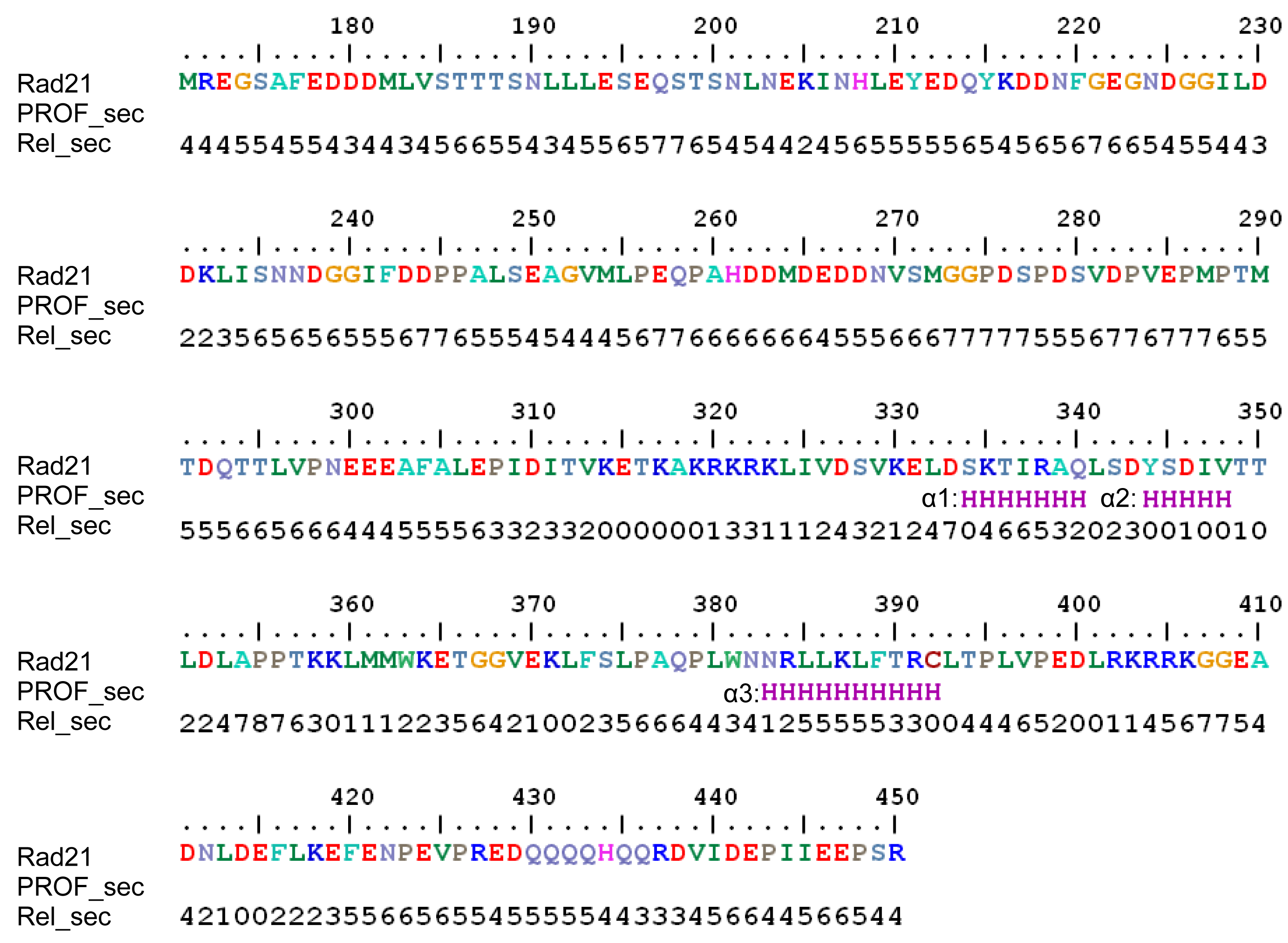

Supplement: Figure S2 — Secondary structure prediction for Rad21 (171–450 aa). Rad21 amino acid sequence is colored based on residue types (e.g. blue for positively charged, red for negatively charged, green for hydrophobic, silver for polar, etc). PROF_sec predicts the secondary structure (H = Helix). Rel_Sec shows the reliability index of the PROF_sec prediction (0 = low, 9 = high). (TIF) [file pone.0069458.s002.tif]

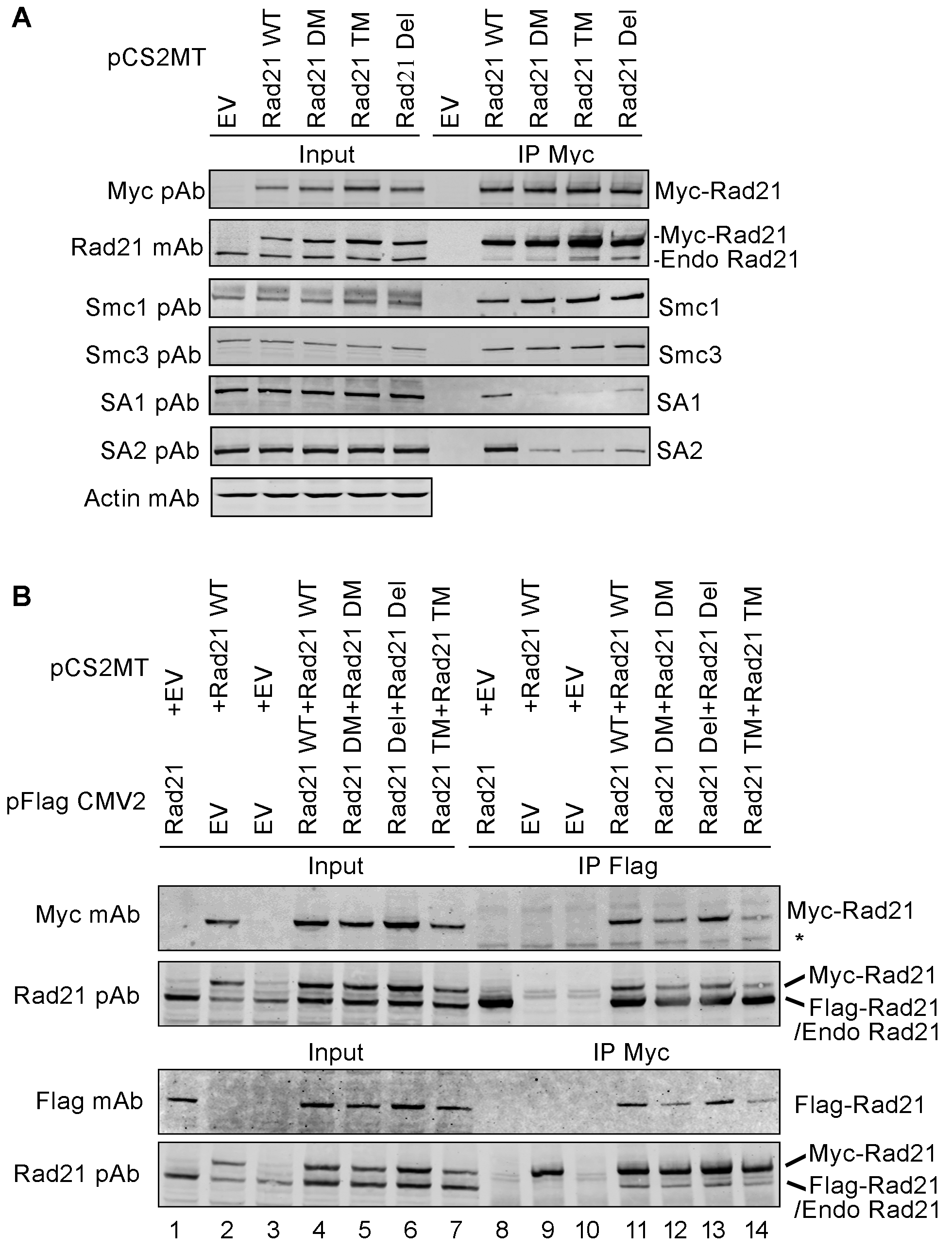

Supplement: Figure S3 — Mutations on middle part SA1/2-binding motif of Rad21 do not affect Rad21-Rad21 interact and co-immunoprecipitate Smc1/3. 293 T cells were transfected with pCM2 MT Rad21 (and pFlag CMV2 Rad21 for (B)). Empty vector was used as control. Co-immunoprecipitation was performed using whole cell lysate. (A) Immunoblotting shows the cohesin core subunits including endogenous Rad21 were co- immunoprecipitated by Myc-Rad21 WT and mutants. (B) Immunoblotting of the co-IP of Flag-Rad21 and Myc-Rad21, which does not affect by the mutation on the middle part of SA1/2 binding motif of Rad21. (TIF) [file pone.0069458.s003.tif]

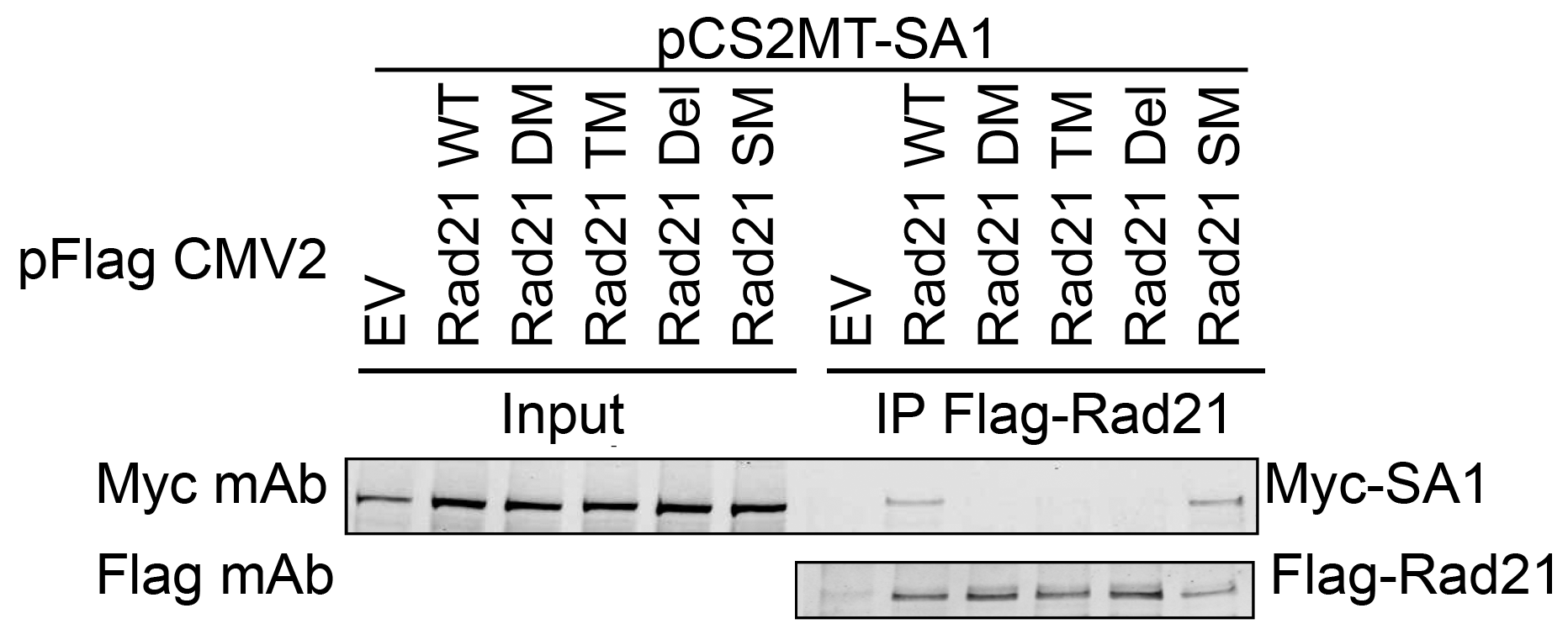

Supplement: Figure S4 — Immunoblotting shows co-immunoprecipitation of Myc-SA1 and Flag-Rad21 WT and mutants. 293 T cells were transfected with pCS2 MT SA1 and pFlag CMV2 Rad21 WT or mutant with mutations on middle part of SA1/2-binding motif. Co-immunoprecipitation was performed using whole cell lysate. EV: empty vector; WT: wild type; SM: L385A; DM: L385A T390A; TM: L385A F389A T390A; Del: del(383–392 aa). (TIF) [file pone.0069458.s004.tif]

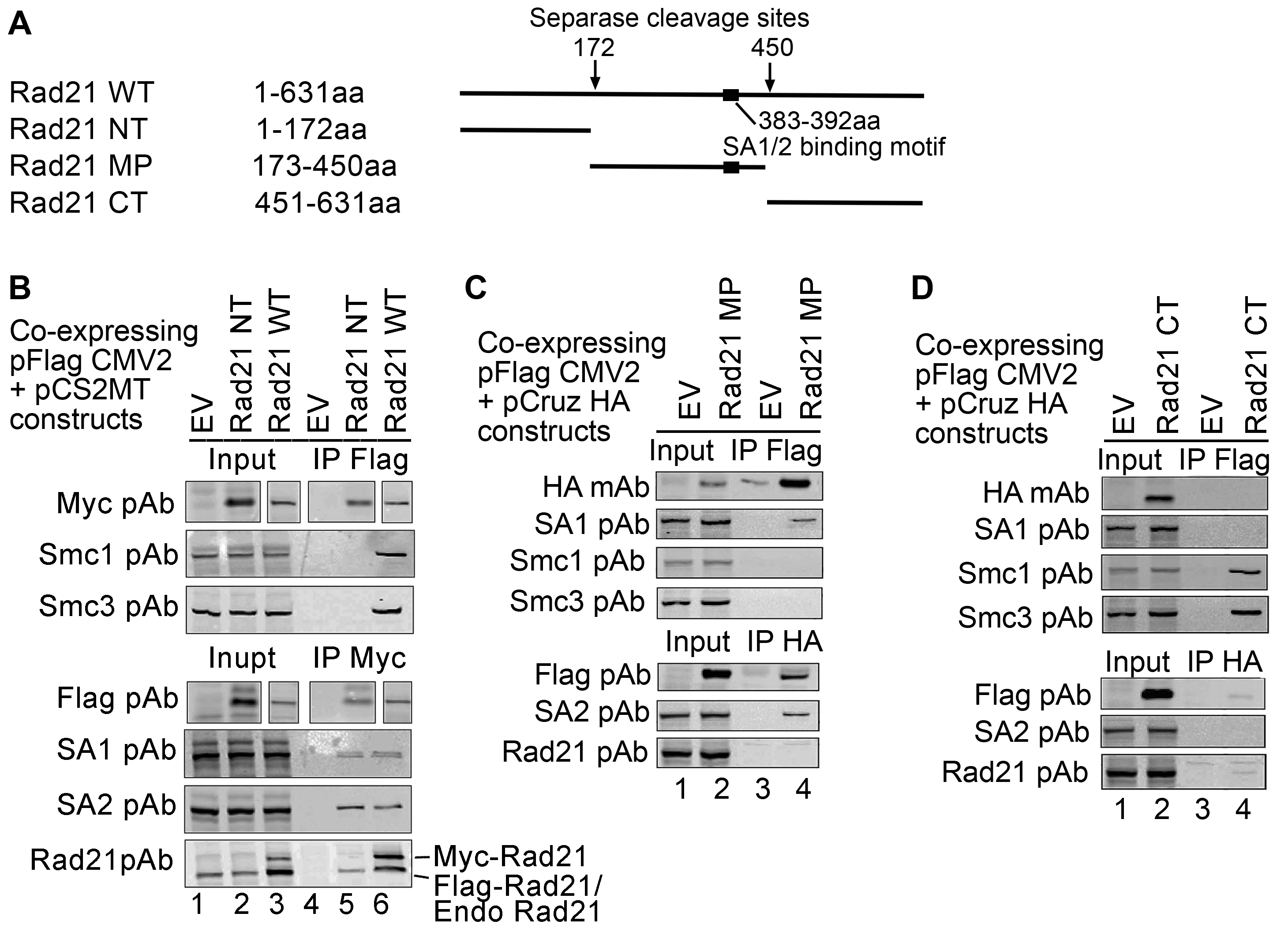

Supplement: Figure S5 — N-terminal Rad21 (1–172 aa) and middle part of Rad21 (173–450 aa) contains SA1/2-binding motif. 293 T cells were transfected with the appropriate plasmids as shown. IP was performed using cell lysates 40 h after transfection. (A) Schematic illustration shows the Rad21 truncated mutants. The Separase cleavage sites at 172 and 450 (arrows) and SA1/2-binding motif at 383–392 aa (rectangle block) are shown. WT: wild type; NT: N-terminus; MP: middle part; CT: C-terminus. (B) Rad21 NT co-immunoprecipitates itself as well as SA1, SA2 and endogenous Rad21 (lane 5), but not Smc1 and Smc3. (C) Flag- and HA-Rad21 MP co-immunoprecipitate each other as well as SA1 and SA2, but fail to co-immunoprecipitate Smc1, Smc3 and Rad21 (lane 4). (D) Flag- and HA-Rad21 CT co-immunoprecipitate Smc1 and Smc3, but fail to co-IP each other and SA1/2 (lane 4). (TIF) [file pone.0069458.s005.tif]
